# ﻿First northwestern Pacific records of the deep-sea cardinalfish *Epigonusglossodontus* (Teleostei, Epigonidae) from the Daito Islands, Japan

**DOI:** 10.3897/zookeys.1231.136445

**Published:** 2025-03-10

**Authors:** Mao Sato, Shohei Ito, Yoshihiro Fujiwara, Keita Koeda

**Affiliations:** 1 Department of Zoology, National Museum of Nature and Science, 4-1-1 Amakubo, Tsukuba, Ibaraki 305-0005, Japan Department of Zoology, National Museum of Nature and Science Tsukuba Japan; 2 FullDepth Co., Ltd., Industrial Liaison and Cooperative Research Center, University of Tsukuba, 1-1-1, Tennodai, Tsukuba, Ibaraki 305-0006, Japan University of Tsukuba Tsukuba Japan; 3 Japan Agency for Marine-Earth Science and Technology (JAMSTEC), 2-15 Natsushima-cho, Yokosuka, Kanagawa 237-0061, Japan Japan Agency for Marine-Earth Science and Technology (JAMSTEC) Yokosuka Japan; 4 Faculty of Science, University of the Ryukyus, 1 Senbaru, Nishihara, Okinawa 903-0213, Japan University of the Ryukyus Nishihara Japan

**Keywords:** Biogeography, fauna, karst, new record, oceanic islands, remotely operated vehicles, taxonomy

## Abstract

The deep-sea cardinalfish *Epigonusglossodontus* Gon, 1985, previously known only from the Hawaiian Islands, was observed using remotely operated vehicles (ROVs) on steep seafloors surrounding Kitadaito and Minamidaito islands, both being oceanic islands belonging to the Daito Islands, Japan in the northwestern Pacific. A total of 44 hours of ROV observations found sparse populations, each of several individuals, around or within small caves, fissures, and recesses, specifically at depths of 340–588 m within the surveyed depth of 284–1009 m. Seven individuals (36.0–114.8 mm standard length) were successfully collected during the ROV observations around the Daito Islands. A subsequent record of the species (97.5 mm standard length) from the Kyushu-Palau Ridge indicated that the species is widely distributed. A glossy bluish-green body color with black-margined scales was revealed by the field observations, the glossy color fading immediately after death.

## ﻿Introduction

Deep-sea cardinalfishes in the genus *Epigonus* (family Epigonidae) include 42 valid species according to Fricke et al. (2024), most having been separated into four species groups i.e., *E.oligolepis* group, *E.constanciae* group, *E.pandionis* group, and *E.telescopus* group (Abramov 1992; Okamoto 2012; Okamoto and Motomura 2013). The *E.oligolepis* group comprises seven species distributed in the central Pacific Ocean (*Epigonusglossodontus* Gon, 1985, *E.carbonarius* Okamoto & Motomura, 2011, and *E.devaneyi* Gon, 1985), Caribbean Sea (*E.oligolepis* Mayer, 1974 and *E.hexacanthus* Okamoto, Baldwin & Long, 2024), and Indian Ocean (*E.exodon* Okamoto & Motomura, 2012 and *E.indicus* Idrees Babu & Akhilesh, 2020). All are characterized by small body size (< 150 mm standard length), large scales with 33–40 pored lateral-line scales, and the absence of a strong opercular spine and ribs on the last abdominal vertebra (Abramov 1992; Okamoto and Motomura 2011, 2012; Idrees Babu and Akhilesh 2020; Okamoto et al. 2024).

Kitadaito and Minamidaito islands, included in the Daito Islands together with Okidaito Island, are oceanic islands characterized by a karst terrain in the northwestern Pacific, about 350 km east of Okinawa Island (Ryukyu Archipelago, southwestern Japan). The respective topographies of the seafloor around Kitadaito and Minamidaito islands are significantly precipitous, each comprising steep slopes and cliffs, with depths exceeding 2000 m at 5 km offshore (Ohde and Elderfield 1992). The geology of Kitadaito Island has been confirmed by drilling as comprising almost entirely carbonate rocks, at least to a depth of 430 m which is the maximum depth sampled (Sugiyama 1934, 1936; Ohde and Elderfield 1992; Suzuki et al. 2006). The island is considered to have been formed by the accumulation of corals during its tectonic movement (accompanied by uplifts and subsidence) from its initial birth as a volcanic island near the Equator to its present location (Klein and Kobayashi 1980; Ohde and Elderfield 1992; Iryu et al. 2023). As on other karst islands, many caves and dolines have formed on land due to the erosion of limestone on both Kitadaito and Minamidaito islands (Yamauchi and Arakaki 1978; Ohde and Elderfield 1992; Knez et al. 2017; Iryu et al. 2023). Such karst features might also exist in the submerged foundations of the islands, but structural details and associated fauna below sea level are poorly understood.

During a deep-sea cave expedition called the “Deep-sea Archaic Refugia in Karst (D-ARK)” project conducted around the Daito Islands in April and May 2024, seven individuals of *E.glossodontus* were captured by three types of remotely operated vehicles (ROVs), as well as many in situ observations made of the species. Originally described from five specimens from the Hawaiian Islands, *E.glossodontus* had not been recorded from any other location to date (Gon 1985; Mundy 2005; Okamoto and Motomura 2011), the specimens from the Daito Islands therefore being the first records of *E.glossodontus* from the northwestern Pacific (including Japanese waters), together with a note on their habitats observed during the ROV surveys. A further specimen record of the species, off the Kita-Koho Seamount (Kyushu-Palau Ridge), about 400 km west of the Daito Islands, was obtained during a subsequent expedition.

## ﻿Material and methods

**Figure 1.**
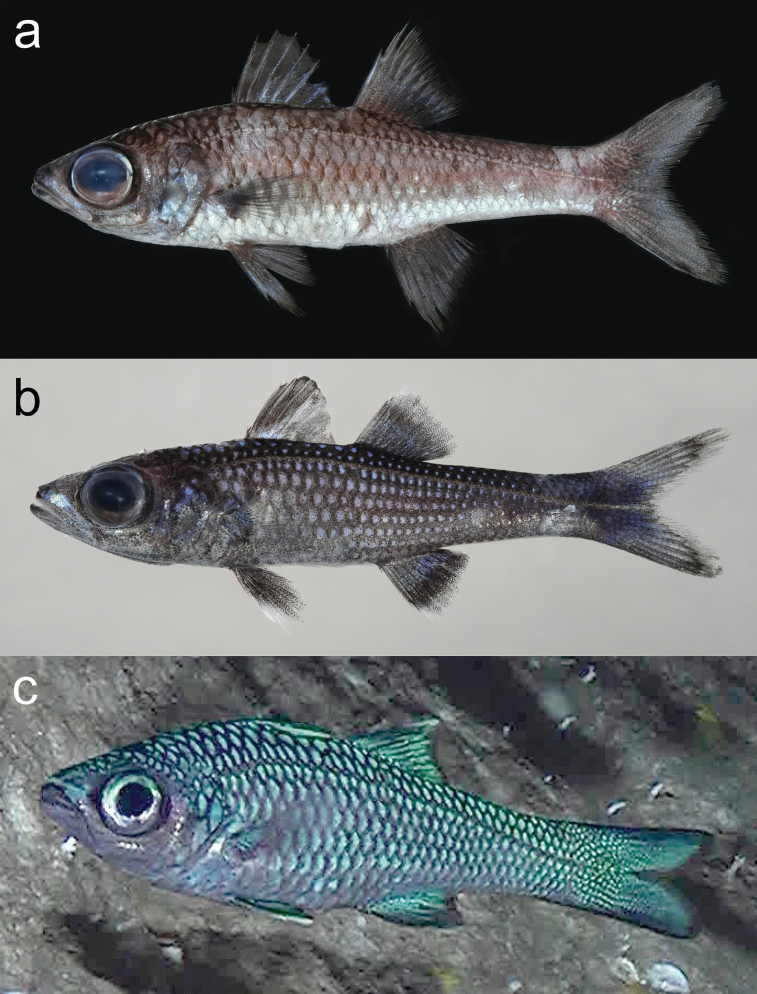
Fresh specimens (**a, b**) and in situ image (**c**) of *Epigonusglossodontus***a**NSMT-P 149549, 114.8 mm SL **b** NSMT-P149551, 54.6 mm SL **c** not collected, image rotated 90° to left.

**Figure 2.**
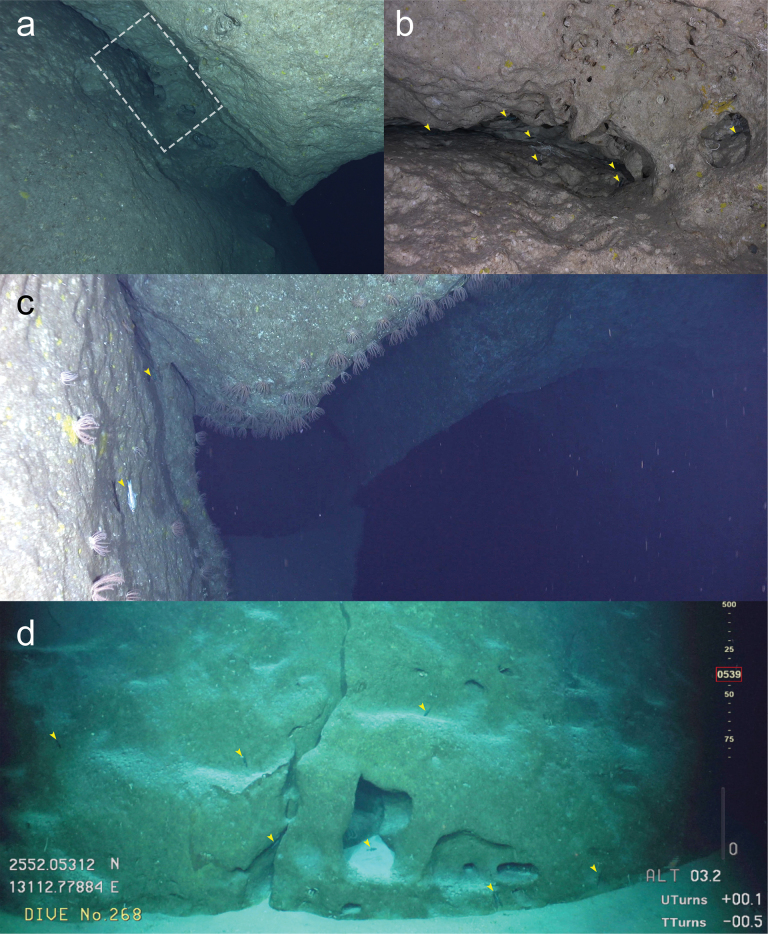
Habitats of *Epigonusglossodontus***a** overhang on steep slope off Kitadaito Island **b** inner part of **a** (indicated by dashed box) **c** cave with branches off Minamidaito Island **d** fissure with small recesses on a wall off Minamidaito Island. Arrowheads indicate *E.glossodontus*.

The specimens examined here have been deposited at NSMT-P (National Museum of Nature and Science) and URIL (University of the Ryukyus, Ichthyological Laboratory). Counts and measurements followed Gon (1985). Standard length is abbreviated as SL. Vertebrae were counted on soft X-ray photographs. Pyloric caeca were counted in three specimens (NSMT-P 149550 and URIL 1428 and 1429) by dissection. The morphological description is based on eight captured specimens (listed below; Fig. 1a, b) and associated underwater video footage, taken by three ROVs (*KM-ROV* and *Crambon* belonging to JAMSTEC; *TripodFinder2* belonging to FullDepth Co., Ltd). Identification of individuals observed in situ using the ROVs was determined based on body proportions, coloration, and the number of pored lateral-line scales (from high resolution images) matching those of the captured individuals. Three representative habitats of *E.glossodontus* near Kitadaito Island (Fig. 2a, b: 25°57.348'N, 131°20.597'E, 343 m depth, observed on 10 May 2024) and Minamidaito Island (Figs 1c, 2c: 25°52.120'N, 131°12.726'E, 398 m depth, 9 May 2024; Fig. 2d: 25°52.531'N, 131°12.779'E, 539 m depth, 5 May 2024) were captured from ROV video footage.

## ﻿Results

### ﻿ Epigonus glossodontus

Taxon classificationAnimaliaPerciformesEpigonidae

Gon, 1985

4A5D42AC-69A9-5F6D-9276-8060BF675399

Figs 1 , 2b, c, d [New standard Japanese name: Horaanahisui-yasemutsu]

Epigonus glossodontus Gon, 1985: 222, figs 1, 2 (holotype locality: off Pearl Harbor, Mamala Bay, Oahu Island, Hawaiian Islands); Okamoto and Motomura 2011: 157–158, figs 2c, g, 4b (Oahu and Molokai islands, Hawaiian Islands).

#### Material examined.

Japan • 1 specimen, 114.8 mm SL; northeast of Minamidaito Island; 25°50.991'N, 131°17.065'E; 537 m depth; 30 April 2024; collected using suction sampler equipped on *KM-ROV*; NSMT-P 149549 • 1, 97.7 mm SL; data as for NSMT-P 149549; URIL 1427 • 1♀, 98.7 mm SL; northeast of Kitadaito Island; 25°57.348'N, 131°20.597'E; 343 m depth; 10 May 2024; suction sampler equipped on *TripodFinder2*; URIL 1428 • 1, 36.0 mm SL; northeast of Kitadaito Island; 25°57.351'N, 131°20.595'E; 342 m depth; 10 May 2024; suction sampler equipped on *TripodFinder2*; NSMT-P 149550 • 1, 58.5 mm SL; south off of Minamidaito Island; 25°48.553'N, 131°15.048'E; 478 m depth; 11 May 2024; suction sampler equipped on *KM-ROV*; URIL 1429 • 1, 54.6 mm SL; data as for URIL 1429; NSMT-P 149551 • 1, 107.3 mm SL; data as for URIL 1429; NSMT-P 149552. HIGH SEA • 1♀, 97.5 mm SL; Kyushu-Palau Ridge, hill west of Kita-Koho Seamount; 26°43.511'N, 135°16.356'E; 562 m depth; 17 May 2024; suction sampler equipped on *Crambon*; NSMT-P 149553.

#### Description.

Meristic and proportional characters are shown in Table 1. Body moderately elongated, compressed, deepest at dorsal-fin origin; nape humped; dorsal and ventral profiles gently curved, slightly concave between first and second dorsal fins. First and second dorsal-fin origins above 5^th^ and 14^th^ lateral-line scales, respectively. Origin of anal fin below middle of second dorsal fin. Origin of pelvic fin at level of dorsal-fin origin; upper base of pectoral fin slightly anterior to point below dorsal-fin origin. Caudal fin deeply forked, with rounded lobes. Pectoral fin rounded, reaching vertical through anus. Respective distal margins of dorsal and anal fins slightly emarginated.

**Table 1.** Counts and measurements of *Epigonusglossodontus*.

		This study	Gon (1985)
		Daito Islands and Kyushu-Palau Ridge	Hawaiian Islands
		(*N* = 8)	(*N* = 5)
Standard length (mm)		36.0–114.8	39.6–78.3
Counts			
	Dorsal fin rays	VII-I, 10	VII-I, 10
	Anal fin rays	II, 9	II, 9
	Principal caudal rays	9+8	9+8
	Pectoral fin rays	17	17
	Pelvic fin rays	I, 5	I, 5
	Pored lateral-line scales	37–38	37–39
	Scales above lateral line	2.5	2.5
	Scales below lateral line	7	7
	Predorsal scales	13–15	13–16
	Branchiostegal rays	7	7
	Gill rakers	5+18–19	5–6+19–21
	Vertebrae	10+15	10+15
	Pyloric caeca	7–8	6–8
Measurements (% of standard length)			
	Head length	32.3–35.0	33.4–38.4
	Head depth	18.7–21.9	-
	Head width	15.2–17.0	-
	Body depth	22.0–26.8	23.1–26.3
	Body width	12.8–16.7	14.6–16.7
	Orbit diameter	12.8–14.7	13.3–15.9
	Interorbital width	7.9–9.7	8.2–9.5
	Eye diameter	10.3–13.3	-
	Snout length	7.1–8.5	6.2–8.7
	Upper jaw length	12.4–15.3	13.2–15.4
	Lower jaw length	14.3–16.9	14.2–17.7
	Postorbital length	10.8–14.1	-
	Caudal peduncle length	26.1–28.4	23.0–25.7
	Caudal peduncle depth	10.3–12.5	8.3–11.4
	First spine length on first dorsal fin	2.3–4.7	2.4–3.0
	Second spine length on first dorsal fin	12.8–15.0	12.7–14.1
	Third spine length on first dorsal fin	14.7–15.8	14.6–15.5
	Second dorsal-fin spine length	10.8–13.2	11.4–12.9
	Longest dorsal ray	17.6–20.0	16.1–21.0
	First spine length on anal fin	1.4–3.4	1.5–2.2
	Second spine length on anal fin	11.4–13.4	11.5–13.1
	Longest anal ray length	16.2–19.3	17.0–18.8
	Pectoral fin length	17.6–21.0	20.4–21.6
	Pelvic fin spine length	11.5–13.8	12.4–13.7
	Pelvic fin length	16.1–19.3	17.7–20.7
	Pre-first dorsal-fin length	35.5–37.8	36.9–39.5
	Pre-second dorsal-fin length	55.0–56.9	56.1–59.2
	Pre-anal fin length	60.1–63.3	62.3–64.2
	Pre-pectoral fin length	31.7–35.8	-
	Pre-pelvic fin length	35.1–38.3	36.1–38.9
	Pre-anus length	53.1–56.9	-
	First dorsal-fin base length	10.6–12.5	11.0–12.6
	Second dorsal-fin base length	11.0–14.1	11.0–12.6
	Anal fin base length	9.2–12.8	10.1–11.5

Head triangular. Snout short, its tip rounded. Eye large, protruding. Pupil circular. Nostrils horizontally level with center of pupil; anterior nostril circular with a short rim directed anteriorly, midway between snout tip and anterior margin of orbit; posterior nostril a vertical slit laterally in front of eye. Mouth small, terminal. Maxillary mustache-like processes absent. Lower jaw slightly projecting; maxilla extending beyond vertical through center of pupil. Conical teeth in a line, decreasing in size posteriorly; upper jaw teeth apparent when mouth closed; two or three large conical teeth projecting anteriorly [not inclined in small specimens (NSMT-P 149550 and 149551 and URIL 1429)] on each side of mandibular symphysis, posteriorly adjacent to 3–6 mid-sized retrorse conical teeth. Mandibular symphysis sunken, toothless. Vomer spoon-shaped, with diamond-shaped head bearing small conical teeth in two (partly three) rows along midline. Palatine thin, with small conical teeth in two rows on entirety. Minute conical teeth scattered on endopterygoid. Tongue broad, with deeply-forked V-shaped teeth patch (opening rostrally) on posterior three-fourths. Posterior margin of preopercle smooth, membranous, covered by ctenoid scales. Opercle with weak ridge hidden under scales. Ribs absent on last abdominal vertebra.

All rays of second dorsal and anal fins branched; last anal ray bifurcating at base. Two uppermost and lowermost pectoral fin rays unbranched. Third spine of first dorsal fin longest. Lateral line generally arched, highest below middle of first dorsal-fin base, anterior (rising) and posterior (lowering) portions straight; last pored lateral-line scale on end of hypural, followed by three or four pored scales and further three small tubular scales on caudal fin. Almost entire head and trunk scaled, except for around nostrils, upper and lower rips, and gular region; all scales ctenoid, these on snout smaller than others; predorsal scales reaching to snout, level with anterior nostrils; one scale row on cheek, encircling ventral and posterior margins of eye. Small cycloid scales covering second dorsal, anal, and caudal fins, except respective distal margins; basal one-third of pectoral fin scaled; pelvic fin without scales, except for base.

***Color***—In life, head and body glossy bluish-green; all scales with black margins; ventral surface whitish. Dorsal, anal, pectoral, and caudal fin scaled areas bluish-green, respective distal margins black; scaleless part of pectoral fin translucent gray. Iris silver, dorsally bluish-green.

Fresh specimens with head and body light coppery-brown, ventrum lighter. All scale posterior margins dark brown, resulting in mottled effect on fins; infraorbital and opercular regions pale bluish. First dorsal fin brown, spines pale bluish; cycloid scales edged with brown, with mottled pattern on scaled areas of second dorsal, anal, and caudal fins. Pelvic and pectoral fins translucent, with dense melanophores.

#### Distribution.

Currently known from Oahu and Molokai islands, Hawaii (Gon 1985; Okamoto and Motomura 2011), Kitadaito and Minamidaito islands in the Daito Islands, Japan, and Kita-Koho Seamount at Kyushu-Palau Ridge (this study).

#### Ecological notes.

During the 44 hours of ROV observations near Kitadaito and Minamidaito islands, at least 122 *E.glossodontus* individuals were sighted at depths between 340–588 m (total depth surveyed 284–1009 m), generally forming sparse schools of several individuals within and around small caves, fissures, and recesses, and slowly swimming a few to some tens of centimeters from the bottom or walls. Swimming was sometimes directed vertically or upside down along the recess walls or ceiling (Figs 1c, 2b, c, d), while a few individuals swam horizontally above the rocky seafloor. Water temperatures in which *E.glossodontus* were observed ranged from 7.9 to 16.7 °C, the temperature range over the entire depth surveyed being 3.8 to 18.2 °C.

The body color of living individuals recorded by the ROVs was overall bluish-green with black-edged scales (Figs 1c, 2c). Although four attempts were made to bring captured individuals to the surface alive, each failed due to decompression, resulting in loss of the bluish-green coloration. Immediately after death, scales were pale blue, but quickly turned coppery-brown (Fig. 1a, b). Dissection of two captured females (NSMT-P 149553 and URIL 1428) revealed well-developed ovaries.

During a subsequent expedition centered on the Kita-Koho Seamount of the Kyushu-Palau Ridge, an ROV dive conducted on a western hill for five hours at depths of 534–778 m, with water temperatures of 5.6–9.3 °C, recorded more than 10 *E.glossodontus* individuals at a site with several recesses at 567 m depth with a temperature of 9.3 °C, where one specimen (NSMT-P 149553) was collected at a later date.

## ﻿Discussion

The present specimens were identified as *Epigonusglossodontus*, a member of the *E.oligolepis* group, based on the following characters: opercular spine weak, poorly ossified; pectoral fin rays 16 or 17; lateral-line scales to end of hypural 37 or 38; scale rows above lateral-line 2.5; gill rakers 23 or 24; two or three large anteriorly projecting teeth on each side of lower jaw symphysis; lingual teeth conical, forming V-shaped apex posteriorly; and pyloric caeca 7 or 8 (Gon 1985; Abramov 1992; Okamoto and Motomura 2011; Okamoto et al. 2024). The meristic and proportional features of the present specimens were all within or overlapped the respective ranges given in the original description of the species, except for a slight difference in caudal peduncle length (Gon 1985; Table 1). Given the overall consistency of other characters, the above difference may be due to intraspecific variation (or different measurement methods). Nevertheless, confirmation with the type series is required for verification. Within the *E.oligolepis* group, *E.glossodontus* has unique dentition i.e., two or three large rostral teeth projecting anteriorly on the lower jar (vs. absent in other species, except *E.exodon*; Gon 1985; Okamoto and Motomura 2011; Okamoto et al. 2024), and a deep V-shaped tooth patch on a broad tongue (vs. shallow V-shaped patch on narrow tongue; Okamoto and Motomura 2012). Previously, six species of *Epigonus* have been recorded from Japanese waters (including the Daito Islands) and the Kyushu-Palau Ridge, specifically *E.atherinoides* (Gilbert, 1905), *E.ctenolepis* Mochizuki & Shirakihara, 1983, *E.denticulatus* Dieuzeide, 1950, *E.fragilis* (Jordan & Jordan, 1922), *E.pectinifer* Mayer, 1974, and *E.elongatus* Parin & Abramov, 1986, none of which are included in the *E.oligolepis* group. All have a greater number of pored lateral-line scales (minimum recorded being 46 in *E.fragilis* vs. 36–39 in *E.glossodontus*) (Okamura et al. 1982; Okamoto 2019; Okamoto and Miyamoto 2022; Okamoto et al. 2024).

Although *E.glossodontus* has been previously recorded only from the Hawaiian Islands (Gon 1985; Mundy 2005; Okamoto and Motomura 2011), the species was the most dominant benthopelagic fish observed during the Daito Islands expedition. It was also confirmed on the Kita-Koho Seamount, indicating a wide distribution in central and northwestern Pacific regions. In fact, within epigonids, *E.fragilis* shows a similar distributional pattern, occurring at depths of several hundred meters on seamounts in both Hawaiian and Japanese waters (Okamoto 2019). The parazenid *Stethopristeseos* Gilbert, 1905 (Parazenidae) is similarly distributed (Koeda et al. 2024).

The type series of *E.glossodontus* was collected by submersible from “small caves in vertical face, 366 m” off Oahu Island (Gon 1985). The Daito Islands’ habitats closely matched the original description, comprising many small caves and recesses on steep rocky slopes (Fig. 2). This underwater landscape may be related to that the islands are made of limestone being susceptible to erosion, but such argument requires further geological investigations. The fact that even the most dominant fish species during the survey period had not been accurately reported in terms of distribution suggests that such challenging environments hold a high potential for further discoveries of not only fish but also many other noteworthy species.

## Supplementary Material

XML Treatment for Epigonus glossodontus

## ﻿Additional information

### Author ORCIDs

Yoshihiro Fujiwara https://orcid.org/0000-0002-1833-1866

Keita Koeda https://orcid.org/0000-0003-3932-3002

### Data availability

All data generated or analyzed during this study are included in this published article
